# Why Animal-Derived Foods Have Been, Are and Will Be Essential for Human Nutrition

**DOI:** 10.3390/nu17193067

**Published:** 2025-09-26

**Authors:** Alicia del Carmen Mondragon Portocarrero, Jose Manuel Miranda

**Affiliations:** Department of Analytical Chemistry, Nutrition and Food Science, School of Veterinary Sciences, University of Santiago de Compostela, 27002 Lugo, Spain; alicia.mondragon@usc.es

## 1. Introduction

Many qualified voices have recently questioned the methods used to establish the relationship between CO_2_ emissions and global warming [1], including leading scientific authorities, such as Nobel Prize winner J.F. Clausser, who opines “global warming, is all a total fabrication by shock journalists and/or dishonest politicians” [2]. Yet, international organizations and national governments with a globalist ideology continue to impose their political agendas over science. Owing to issues arising from certain political agendas, animal-based foods are being attacked unfairly from various angles. For example, political organizations such as the European Union are passing regulations that significantly hinder livestock farming and fishing.

The European Green Deal [3] and Common Agricultural Policy [4] reforms aim to achieve substantial environmental and climate objectives, requiring changes in agricultural practices. As a result of the application of such policies, a marked decrease in the production of animal products and a subsequent increase in market prices have occurred [5,6]. This has caused an significant reduction in the EU’s agricultural production and reduced the overall market competitiveness of EU nations [5].

On the other hand, there is also a trend toward encouraging the population to reduce their consumption of animal-based foods and replace them with plant-based foods, fewer conventional foods, or other less conventional foods, such as cultured meat. These changes legitimize not necessarily balanced diets, which can lead to lower growth parameters and malnutrition [7]. In addition, these changes in dietary habits seriously damage small food producers from rural communities, while they provide good profits for large multinational companies and ultra-processed food producers [8]. This has caused rural areas to become rapidly depopulated, leading to job losses and the loss of cultural heritage, as well as contributing to environmental disasters such as the large number of forest fires that occurred in Spain this year [9].

Based on this, we considered it necessary to provide a platform for authors who wish to publish works on the benefits animal-based foods can have for human health. This Special Issue includes a total of eight published articles, two of which are experimental articles (Contributions 1 and 8), while the other six are bibliographic reviews (Contributions 2–7). One of the articles addresses the importance of salmon in various forms as a source of vitamin D for young women (Contribution 1). This study concluded that increasing salmon sausage intake might aid in slowing the natural decline of vitamin D in young women in autumn.

Contribution 2 evaluates the relevance of eggs in modulating the human gut microbiome, promoting the production of gastrointestinal-related metabolites, and mediating inflammation. This systematic literature review revealed conflicting results regarding egg consumption and most gastrointestinal outcomes, highlighting that future studies are needed to explore the links between habitual egg intake and plasma trimethylamine N-oxide levels, microbial diversity, and inflammation.

Two review articles evaluated the relevance of terrestrial animal source foods (TASFs), the former as a source of nutrients and bioactive compounds for the general population (Contribution 3) and the latter as food for vulnerable populations (Contribution 4). Contribution 3 concluded that it is necessary to include meat from hunting and wildlife farming and insects in global food composition databases, as the literature on these foods is scant. The authors also conclude that scarce research has focused on TASFs in low- and middle-income countries, highlighting the need for further exploration of how intrinsic animal characteristics and livestock production system characteristics impact the nutritional value of TASFs. Contribution 4 remarked that sufficient evidence supports an important role for TASFs in vulnerable population health during the course of life.

In some countries, there is a growing trend among parts of the population to replace animal milk with plant-based substitutes [10]. Contribution 5 concludes that, from the perspective of their effects on the intestinal microbiota, milks of animal origin are more beneficial for human health than their vegetable substitutes are. With respect to animal-derived milk, Contribution 6 reviews the properties of lactoferrin, a multifunctional glycoprotein naturally found in colostrum and milk. Lactoferrin is considered a promising therapeutic protein to combat viral infections by hindering the viral entry process at the beginning of the viral life cycle, and it is an effective antiviral protein even during early infection.

Bioactive compounds other than lactoferrin can be found in colostrum (Contribution 7), such as immunoglobulins, oligosaccharides, and lysozyme. Colostrum also contains growth factors that contribute to wound healing and muscle and bone development and support growth in children. Additionally, the molecular mechanisms involved in these processes are explored, highlighting the roles of growth factors in cell proliferation, tissue regeneration, and the regulation of immune responses. Contribution 7 examines the concentrations of growth factors in bovine colostrum, as well as their benefits, molecular mechanisms, and results from clinical studies of such growth factors.

Finally, Contribution 8 presents a randomized controlled trial investigating how the sustained consumption of bovine, caprine, or ovine milk influences digestion, nutrition, and metabolism in older women. These findings could inform dietary recommendations for older women and facilitate the development of targeted functional food products.
